# Hip Osteoarthritis in Dogs: A Randomized Study Using Mesenchymal Stem Cells from Adipose Tissue and Plasma Rich in Growth Factors

**DOI:** 10.3390/ijms150813437

**Published:** 2014-07-31

**Authors:** Belen Cuervo, Monica Rubio, Joaquin Sopena, Juan Manuel Dominguez, Jose Vilar, Manuel Morales, Ramón Cugat, Jose Maria Carrillo

**Affiliations:** 1 Department of Animal Medicine and Surgery, CEU Cardenal Herrera University, C/Tirant lo Blanc, 7, 46115 Alfara del Patriarca, Valencia, Spain; E-Mails: mrubio@uch.ceu.es (M.R.); jsopena@uch.ceu.es (J.S.); jcarrillo@uch.ceu.es (J.M.C.); 2 Garcia Cugat Foundation CEU UCH Chair of Medicine and Regenerative Surgery, 08006 Barcelona, Spain; E-Mail: ramon.cugat@sportrauma.com; 3 Department of Animal Medicine and Surgery, University of Cordoba, 14071 Cordoba, Spain; E-Mail: pv2dopej@uco.es; 4 Department of Animal Medicine and Surgery, University of Las Palmas de Gran Canaria, 35413 Las Palmas de Gran Canaria, Spain; E-Mails: jvilar@dpat.ulpgc.es (J.V.); mmorales@dpat.ulpgc.es (M.M.); 5 Artroscopia GC, Plaza Alfonso Comin, 12, 08023 Barcelona, Spain

**Keywords:** adipose derived mesenchymal stem cells, plasma rich in growth factors, osteoarthritis, dog, hip

## Abstract

Purpose: The aim of this study was to compare the efficacy and safety of a single intra-articular injection of adipose mesenchymal stem cells (aMSCs) *versus* plasma rich in growth factors (PRGF) as a treatment for reducing symptoms in dogs with hip osteoarthritis (OA). Methods: This was a randomized, multicenter, blinded, parallel group. Thirty-nine dogs with symptomatic hip OA were assigned to one of the two groups, to receive aMSCs or PRGF. The primary outcome measures were pain and function subscales, including radiologic assessment, functional limitation and joint mobility. The secondary outcome measures were owners’ satisfaction questionnaire, rescue analgesic requirement and overall safety. Data was collected at baseline, then, 1, 3 and 6 months post-treatment. Results: OA degree did not vary within groups. Functional limitation, range of motion (ROM), owner’s and veterinary investigator visual analogue scale (VAS), and patient’s quality of life improved from the first month up to six months. The aMSCs group obtained better results at 6 months. There were no adverse effects during the study. Our findings show that aMSCs and PRGF are safe and effective in the functional analysis at 1, 3 and 6 months; provide a significant improvement, reducing dog’s pain, and improving physical function. With respect to basal levels for every parameter in patients with hip OA, aMSCs showed better results at 6 months.

## 1. Introduction

Osteoarthritis (OA) is a very common degenerative disease affecting the articular cartilage in both human [1] and veterinary medicine [2,3]. This condition affects 15% of the world population, amounting to colossal health-care costs [4], and has a great impact on a patient’s quality of life [5].

The current therapeutic approaches focus on preventing or at least delaying the structural and functional changes of OA. The use of stem cell-based therapies and Platelet Rich Plasma (PRP) for repair and regeneration in OA has become a new avenue of treatment as an alternative to the more aggressive therapies [6,7,8,9,10].

Stem cells may be of embryonic origin (ESCs) or adult (ASCs). Adult stem cells have a much lower capacity than embryonic stem cells to self-renew and differentiate along multiple lineage pathways. However, adult stem cells are immunocompatible, and their use is not restricted by the ethical concerns associated with embryo-derived cells. Apart from the ethical concerns embryonic cells are known to raise, they have also been shown to demonstrate uncontrolled growth [11,12]. In contrast, adult stem cells, including mesenchymal stem cells (MSCs), are a very good option as they are present in a number of postnatal organs and connective tissues, and are not subject to the same restrictions as the embryonic cells [13]. Mesenchymal stem cells can be easily isolated from many adult tissues such as bone marrow, placenta [14], umbilical cord [15], skeletal muscle [16], synovium [17], synovial fluid [18], and adipose tissue [8,19] among others. An increasing amount of studies are using adipose tissue derived mesenchymal cells (aMSCs) in the treatment of OA, as large quantities are easily harvested with little donor site morbidity or patient discomfort, as well as demonstrating the ability to differentiate into chondrocytes, osteocytes and adipocytes [20]. It has been shown that autologous stem cells have an affinity for damaged joint tissue; recent studies have confirmed that stem cells have the ability to localize and participate in the repair of damaged joint structures [21]. Recently, it was published that aMSC therapy was found to be an appropriate treatment for hip joints in dogs, improving the dog’s gait and ability to live a more normal life [22].

One of the most popular methods used to biologically enhance healing in the fields of orthopedic surgery and sports medicine includes the use of autologous blood products, particularly, PRP [23]. PRP is defined as the volume of autologous plasma having a platelet concentration over baseline [24,25,26], and is also referred to as plasma rich in growth factors (PRGF), PRP-gel, platelet gel or platelet clot [27,28,29]. Under normal circumstances, platelets are the first cell type to arrive at the tissue injury site and are particularly active in the early inflammatory phases [30]. Several studies describe the use of PRP as an effective and safe method in the treatment of pain and joint dysfunction in OA. The use of PRGF (PRGF-Endoret; BTI Biotechnology Institute, Vitoria, Spain), an autologous PRP characterized by the absence of leukocytes and proinflammatory cytokines and the presence of a specific dose of platelets and growth factors [28], has been demonstrated to achieve significant improvement in humans with knee OA [5,31] and also in dogs with OA [32].

Currently, the effectiveness of these two therapies has been demonstrated for the treatment of OA, however in the literature, there are no studies comparing the results of these two treatments with each other. The aim of this study was to compare the efficacy and safety in a randomized, clinical trial of a single intra-articular injection of adipose mesenchymal stem cells (aMSCs) (Dog-Stem, Fat-Stem, Aalst, Belgium) *versus* a single intra-articular injection of plasma rich in growth factors (PRGF) (PRGF-Endoret, BTI Biotechnology Institute, Vitoria-Gasteiz, Araba, Spain) as a treatment for reducing symptoms in dogs with hip osteoarthritis (OA), assessing the effectiveness of each treatment and determine the advantages and disadvantages of each.

## 2. Results and Discussion

### 2.1. Results

#### 2.1.1. Animal Population

**Figure 1:**
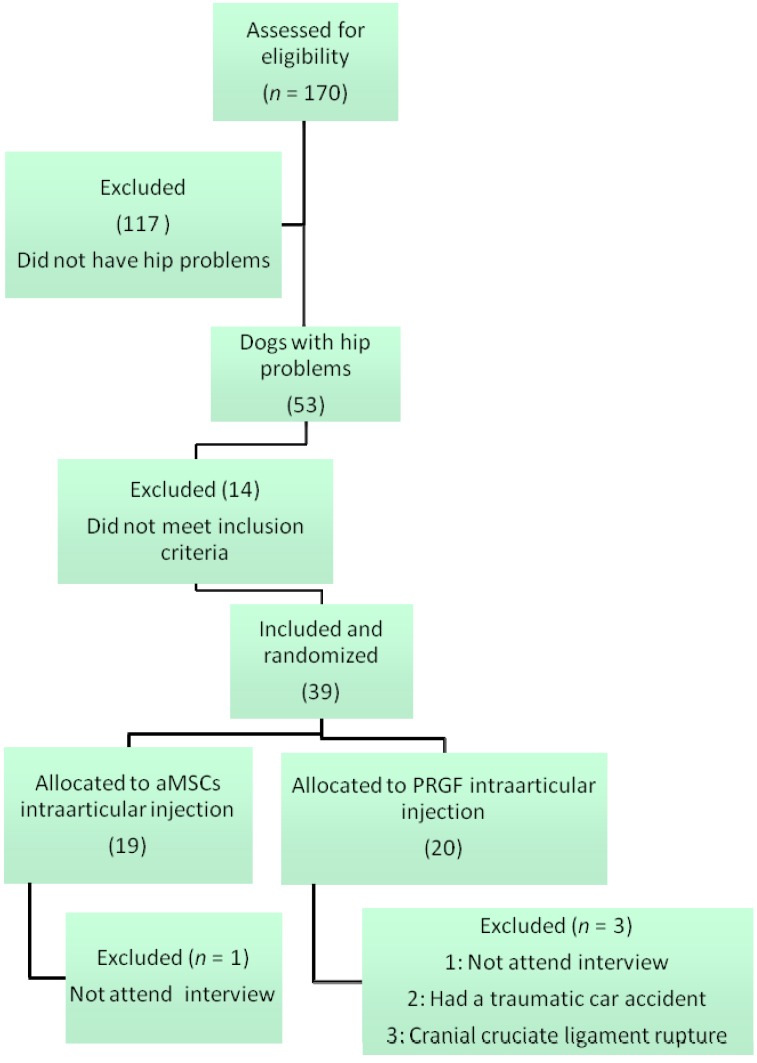
Enrollment and outcomes.

A total of 70 patients with lameness in the legs were assessed for eligibility. Only 53 of them presented a hip problem, from which 14 were not included, as they did not meet the inclusion criteria. Finally, 39 animals were screened, and randomized into their corresponding group. Four were lost to follow up, 1 in the aMSCs group and 3 in the PRGF group (Figure 1).

Animals were recruited from January 2002 to January 2003. Animals included in the study, attended clinic visits at the time of randomization (baseline) and at 1, 3 and 6 months after receiving the treatment.

The mean age and weight of the patients was 53 ± 43 months (age range: 8–135 months), and 34.9 ± 12.8 kg (weight range: 18.3–66.2 kg) in the aMSCs group and 93 ± 35.5 (age range: 18–66) and 36.5 ± 10.6 kg (weight range 20–62.8 kg), in the PRGF group. Twenty-four of the patients were male, 10 in aMSCs and 14 in PRGF, and 11 were female, 8 in the aMSCs group and 3 in the PRGF group (Table 1).

#### 2.1.2. Pain Assessment

**Figure 2:**
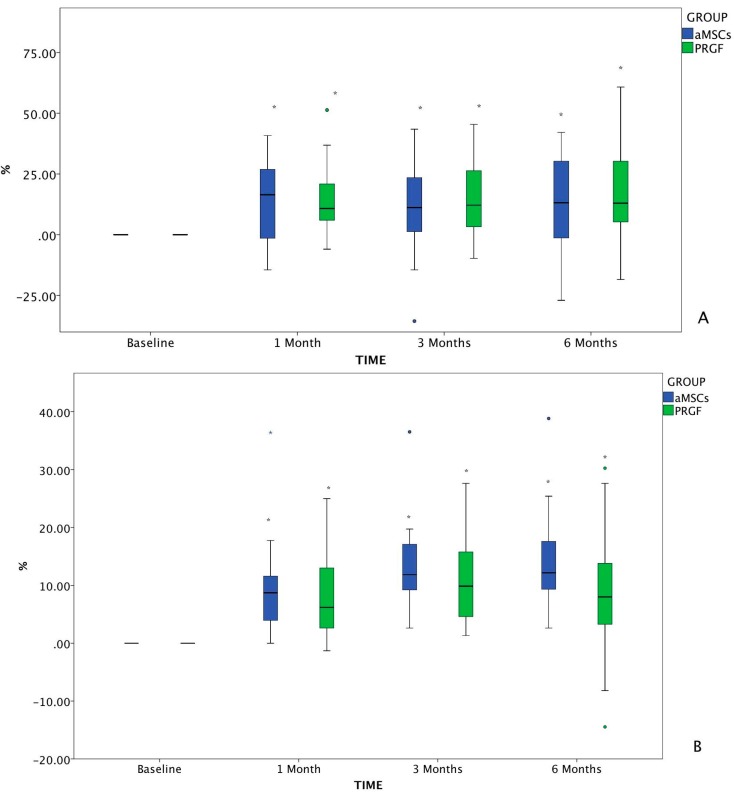
Changes in VAS from baseline value assessed by the owner (**A**) and the investigator (**B**). The changes in VAS which differed significantly with baseline (*p* < 0.05) are marked as *. The circles, both green and blue, correspond to anomaly/outlier data included in the statistical study. The blue asterisk corresponds to an outlier, to which the Dixon Q test was applied in order to confirm deletion of the value from the database.

The VAS results were calculated as % change from baseline at all treatment time points (Figure 2). Results of pain assessment (VAS scores) measured at baseline and outcomes for the entire population are summarized in Table 2.

**Table 1:** Baseline characteristics of patients assessed.

Characteristic	aMSCs	PRGF	*p*-Value
Age (months)	53 ± 43 (8–135)	93 ± 35.5 (18–66)	0.007
Weight	34.9 ± 12.8 (18.3–66.2)	36.5 ± 10.6 (20–62.8)	0.805
Gender (male-female)	10-8	14-3	0.201
Radiographic Osteoarthritis degree Bioarth Score *	15.47 ± 6.02 (13.37–17.57)	17.36 ± 3.8 (16.07–18.65)	0.259
Functional limitation	5.88 ± 2.83 (2–11)	8.22 ± 3.39 (1–14)	0.003
Joint mobility	4.18 ± 0.63 (3–5)	3.92 ± 0.84 (2–6)	0.131
Muscle atrophy	1.05 ± 0.55572	1.0526 ± 0.62126	0.890
Owner Vas Score	28.29 ± 14.49 (6.57–72.37)	42.55 ± 20.52 (9.21–72.37)	0.023
Patients	18	17	

Quantitative variables are expressed as mean ± standard deviation (range). *p* < 0.05 is considered statistically significant. * The Bioarth score is an index of severity for hip mobility that includes 4 subscales (radiographic findings (0–21 points), functional limitation (0–23), joint mobility (0–7 points) and muscle atrophy (0–2 points).

In the owner’s pain assessment, significant differences were observed in the aMSCs and PRGF groups between baseline and 1, 3 and 6 months post-treatment, with no statistically significant differences between them.

In the investigator assessment, significant differences were observed in both groups between baseline and all other follow-up time points. Comparing both treatments, there were only differences at 6 months post-infiltration, where patients treated with aMSCs showed more pain relief than those treated with PRGF.

**Table 2:** The mean VAS (±SD) assessed by the dog owners and by the investigator at each time point. For statistical purposes, the Kruskal Wallis Test was applied (Spss Statistics for MAC, version 20, IBM, Madrid, Spain). Differences between groups are shown as *p*-value between groups. *p* <0.05 is considered statistically significant.

Variable	Time	Group	*N*	Mean	Std. Deviation	95% Confidence Interval for Mean		Minimum	Maximum	*p*-Value between Groups
						Lower Bound	Upper Bound			
Owner VAS	baseline	aMSCs	34	28.6353	14.33904	23.6322	33.6384	6.57	53.94	0.001
		PRGF	36	42.9756	20.61379	36.0009	49.9503	9.21	72.37	
		Total	70	36.0103	19.13089	31.4487	40.5719	6.57	72.37	
	1 month	aMSCs	34	15.4312	11.03277	11.5817	19.2807	0.00	48.68	0.000
		PRGF	36	27.5978	13.61288	22.9918	32.2037	5.92	59.21	
		Total	70	21.6883	13.77408	18.4040	24.9726	0.00	59.21	
	3 months	aMSCs	34	17.5500	15.87660	12.0104	23.0896	1.31	57.89	0.014
		PRGF	36	27.4081	16.72219	21.7501	33.0660	4.60	72.37	
		Total	70	22.6199	16.94175	18.5802	26.6595	1.31	72.37	
	6 months	aMSCs	30	16.8620	13.55143	11.8018	21.9222	2.63	51.31	0.046
		PRGF	34	24.6088	16.43983	18.8727	30.3450	0.00	56.58	
		Total	64	20.9775	15.53332	17.0974	24.8576	0.00	56.58	
Veterinarian VAS	baseline	aMSCs	34	23.8200	10.77498	20.0604	27.5796	6.57	50.66	0.000
		PRGF	36	32.9692	9.88435	29.6248	36.3136	10.60	48.68	
		Total	70	28.5253	11.23820	25.8456	31.2049	6.57	50.66	
	1 month	aMSCs	34	14.0894	6.38365	11.8621	16.3168	6.57	32.89	0.000
		PRGF	36	24.8964	7.74936	22.2744	27.5184	8.00	42.76	
		Total	70	19.6473	8.91895	17.5206	21.7739	6.57	42.76	
	3 months	aMSCs	34	10.8635	5.89049	8.8082	12.9188	3.95	26.31	0.000
		PRGF	36	22.0383	7.50788	19.4980	24.5786	6.00	40.79	
		Total	70	16.6106	8.76544	14.5205	18.7006	3.95	40.79	
	6 months	aMSCs	32	9.5306	5.77949	7.4469	11.6144	3.95	29.60	0.000
		PRGF	34	24.8297	8.75538	21.7748	27.8846	6.00	50.00	
		Total	66	17.4120	10.68689	14.7848	20.0391	3.95	50.00	

#### 2.1.3. Degree of Osteoarthritis Based on the Radiographic Findings

When OA degree was categorized at baseline, 3 patients had mild OA, 2 moderate OA and 13 severe OA in the aMSCs group. However in the PRGF group, 5 patients presented moderate OA and 14 severe OA. There were no significant differences between groups. Except for 1 dog in the aMSCs group that at one month presented severe OA, the rest of the dogs’ radiographic scores at 6 months were the same as the scores at baseline in both groups.

#### 2.1.4. Bioarth Scale Assessment

Global Scores of all the variables assessed with the Bioarth scale for hip OA in both groups at baseline and its outcomes are summarized in Table 3.

**Table 3:** Mean ± SD of the functional limitation, joint mobility and range of motion, at baseline, one, three and six months after treatment. Kruskal-Wallis results between groups. * Differences with baseline.

Variable	Time	Group	*N*	Mean		Std. Deviation	95% Confidence Interval for Mean		Minimum		Maximum	*p*-Value between Groups	
							Lower Bound	Upper Bound					
OA degree	baseline	aMSCs	34	15.47		6.02	13.37	17.57	3.00		21.00	0.259	
		PRGF	36	17.36		3.80	16.07	18.65	10.00		21.00		
		Total	70	16.44		5.06	15.24	17.65	3.00		21.00		
	1 month	aMSCs	34	15.62		6.11	13.49	17.75	3.00		21.00	0.339	
		PRGF	36	17.36		3.80	16.07	18.65	10.00		21.00		
		Total	70	16.51		5.10	15.30	17.73	3.00		21.00		
	3 months	aMSCs	34	15.74		6.16	13.59	17.88	3.00		21.00	0.402	
		PRGF	36	17.39		3.81	16.10	18.68	10.00		21.00		
		Total	70	16.59		5.12	15.36	17.81	3.00		21.00		
	6 months	aMSCs	32	15.75		6.44	13.43	18.07	3.00		21.00	0.714	
		PRGF	34	17.18		3.82	15.84	18.51	10.00		21.00		
		Total	66	16.48		5.26	15.19	17.78	3.00		21.00		
Functional Limitation	baseline	aMSCs	34	5.88		2.83	4.90	6.87	2.00		11.00	0.003	
		PRGF	36	8.22		3.39	7.08	9.37	1.00		14.00		
		Total	70	7.09		3.32	6.29	7.88	1.00		14.00		
	1 month	aMSCs	34	2.85 *		1.94	2.18	3.53	0.00		7.00	0.009	
		PRGF	36	4.56 *		2.75	3.62	5.49	0.00		10.00		
		Total	70	3.73		2.52	3.13	4.33	0.00		10.00		
	3 months	aMSCs	34	2.26 *		1.75	1.66	2.87	0.00		6.00	0.181	
		PRGF	36	3.33 *		2.95	2.34	4.33	0.00		12.00		
		Total	70	2.81		2.48	2.22	3.41	0.00		12.00		
	6 months	aMSCs	30	1.80 *		1.54	1.22	2.38	0.00		5.00	0.004	
		PRGF	34	3.71 *		2.76	2.74	4.67	0.00		9.00		
		Total	64	2.81		2.45	2.20	3.42	0.00		9.00		
Joint Mobility	baseline	aMSCs	34	4.18		0.63	3.96	4.39	3.00		5.00	0.131	
		PRGF	36	3.92		0.84	3.63	4.20	2.00		6.00		
		Total	70	4.04		0.75	3.86	4.22	2.00		6.00		
	1 month	aMSCs	34	2.4 *		1.23	1.98	2.84	0.00		4.00	0.057	
		PRGF	36	2.97 *		0.88	2.68	3.27	1.00		4.00		
		Total	70	2.70		1.09	2.44	2.96	0.00		4.00		
	3 months	aMSCs	34	1.38 *		1.52	0.85	1.91	0.00		4.00	0.000	
		PRGF	36	2.75 *		1.02	2.40	3.10	1.00		4.00		
		Total	70	2.09		1.45	1.74	2.43	0.00		4.00		
	6 months	aMSCs	32	1.09 *		1.40	0.59	1.60	0.00		4.00	0.000	
		PRGF	34	2.82 *		1.00	2.47	3.17	1.00		4.00		
		Total	66	1.98		1.48	1.62	2.35	0.00		4.00		
Muscle Perimeter (cm)	baseline	aMSCs	34		32.56	5.37	30.69	34.44	25.00	47.00			0.609
		PRGF	36		32.42	3.07	31.38	33.46	27.50	41.00			
		Total	70		32.49	4.31	31.46	33.52	25.00	47.00			
	1 month	aMSCs	34		33.51 *	4.97	31.78	35.25	27.00	45.00			0.005
		PRGF	36		32.56	3.24	31.46	33.65	26.00	40.00			
		Total	70		33.02	4.17	32.03	34.01	26.00	45.00			
	3 months	aMSCs	34		33.26 *	4.40	31.73	34.80	28.00	43.00			0.984
		PRGF	36		32.34	3.33	31.21	33.46	25.00	41.00			
		Total	70		32.79	3.89	31.86	33.71	25.00	43.00			
	6 months	aMSCs	32		33.98 *	4.51	32.36	35.61	27.00	43.00			0.000
		PRGF	34		32.50	3.35	31.33	33.67	26.00	40.00			
		Total	66		33.22	4.00	32.24	34.20	26.00	43.00			
Range of Motion	baseline	aMSCs	34		64.12	12.06	59.91	68.33	40.00	90.00			0.000
		PRGF	36		75.61	12.22	71.48	79.75	54.00	95.00			
		Total	70		70.03	13.37	66.84	73.22	40.00	95.00			
	1 month	aMSCs	34		96.65 *	13.21	92.04	101.26	60.00	125.00			0.002
		PRGF	36		87.69 *	11.71	83.73	91.66	55.00	105.00			
		Total	70		92.04	13.16	88.90	95.18	55.00	125.00			
	3 months	aMSCs	34		103.71 *	12.26	99.43	107.98	60.00	125.00			0.000
		PRGF	36		92.67 *	11.35	88.83	96.51	68.00	115.00			
		Total	70		98.03	12.96	94.94	101.12	60.00	125.00			
	6 months	aMSCs	32		107.09 *	8.56	104.01	110.18	90.00	122.00			0.000
		PRGF	34		90.50 *	11.24	86.58	94.42	60.00	105.00			
		Total	66		98.55	12.99	95.35	101.74	60.00	122.00			

##### Functional Limitation

**Figure 3:**
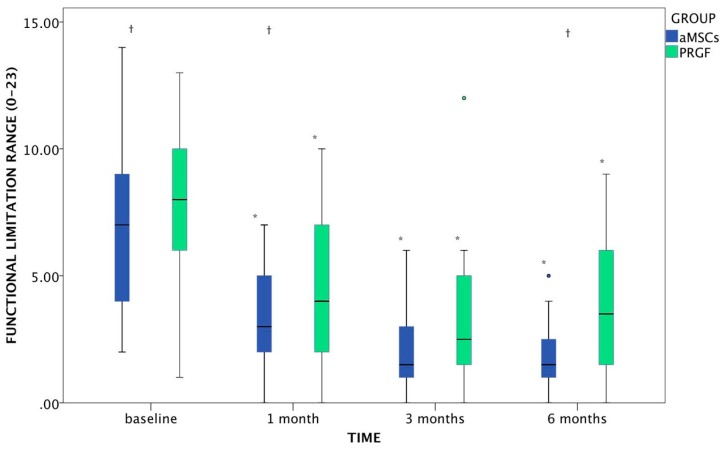
Evolution of functional limitation (range: 0–23) in dogs after aMSCs or PRGF treatment at the 6-months follow up period. The changes which differed significantly with baseline (*p* < 0.05) are marked as *. Statistically significant differences between groups are marked as †. Circles, both green and blue, correspond to outlier included in the statistical study.

Both groups showed significant improvement in functional limitation at all the times evaluated. There were significant differences between groups at baseline (*p* = 0.003), where PRGF presented more limitation to normal life than the aMSCs group; at 1 month this difference was maintained (*p* = 0.009), but at 3 months the quality of life was similar in both groups (*p* = 181), moreover at 6 months aMSCs continued with a better score in functional limitation, whereas PRGF started to decrease (*p* = 0.004) (Figure 3).

##### Joint Mobility

In the overall joint mobility, both groups improved significantly, showing differences to baseline at each time point. In addition, by 3 and 6 months, joint mobility in the aMSCs group was significantly higher than the PRGF group (*p* < 0.001 at both times).

Regarding the range of motion (ROM), significant improvement was noted in both groups, obtaining significant differences compared to baseline.

**Figure 4:**
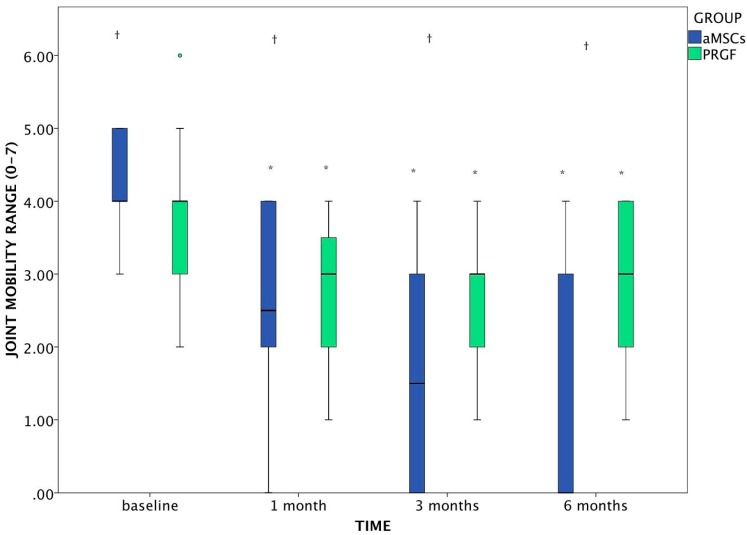
Evolution of joint mobility (range: 0–7) in dogs after aMSCs or PRGF treatment at the 6-months follow up period. The changes which differed significantly with baseline (*p* < 0.05) are marked as *. Statistically significant differences between groups are marked as †. The green circle is an outlier included in the statistics study.

Regarding the differences between groups there were significant differences from baseline (*p* = 0.001) with greater range of motion noted in the PRGF group, but since the first visit, animals from the aMSCs group obtained better results at 1, 3 and 6 months (*p* = 0.002; *p* < 0.001; *p* < 0.001; respectively), which indicates that the aMSCs group had a much greater increase in range of motion (Figure 4).

##### Muscle Atrophy

In the muscle atrophy assessment, neither of the two groups demonstrated significant differences at the different control periods. Compared with baseline, aMSCs group presented significant differences at 1 (*p* = 0.002), 3 (*p* = 0.048) and 6 (*p* < 0.001) months. In contrast, in the PRGF group muscle mass remained constant throughout the study.

#### 2.1.5. Overall Satisfaction with Treatment

Fourteen and 9 animals from the aMSCs and PRGF groups, respectively, were treated with NSAIDs and nutraceuticals. Regarding treatment efficacy after the intra-articular injection of aMSCs or PRGF, satisfaction was high in both groups.

According to the dog owners, quality of life improved for patients in both groups between baseline and 6 months in aMSCs (*p* = 0.004) and in PRGF (*p* = 0.001) groups. However, statistically significant differences between groups were shown at 6 months, where the aMSCs group scored higher than the PRGF group (*p* = 0.028).

In all cases, at the end of the treatment, owners were asked if they would use the same treatment again for their pets, and all of them responded positively.

#### 2.1.6. Safety Assessment

Two adverse events, one in the aMSCs group and one in the PRGF group, were reported during the study. The adverse effect was mild and no differences were observed between groups (*p* = 0.671). The owners of the two animals reported pain post-injection and prolonged the NSAID treatment for 2 days in the aMSCs group, and 4 days in the PRGF group, with no significant differences between them.

#### 2.1.7. Requirement of Rescue Analgesia

At 3 months, 1 dog in the aMSCs and 2 in the PRGF group were given meloxicam as rescue analgesic, without significant differences between them (*p* = 0.365), and at 6 months none of the dogs required analgesic in the aMSCs group and 2 animals did in the PRGF group, also without significant differences (0.064).

### 2.2. Discussion

The results from this randomized trial show that a single intra-articular injection of aMSCs is significantly more effective than one intra-articular injection of PRGF in reducing pain and improving functional limitation and quality of life in dogs with hip OA. To the author’s knowledge, the present study is the first that showed that both treatments reported good results from the first month post-treatment and up to six months, although better results were subjectively observed at 6 months in patients treated with aMSCs. The data obtained in the present study supports the use of these therapies as a treatment for OA.

Among the wide variety of sources for adult mesenchymal cells [33] in this study MSCs were obtained from adipose tissue due to the ease of extraction and the absence of complications associated with this process [13,34]. Likewise, adipose tissue is a very rewarding source of mesenchymal cells, which are easily harvested and expanded in culture and have a high proliferation rate [19], capable of differentiation into chondrocytes, osteocytes and adipocytes [20].

The main adipose tissue harvest areas in previous studies in dogs are the falciform ligament [35], lateral thoracic area [36], caudal scapular region [37], intra-abdominal or subcutaneous fat obtained during ovariohysterectomy surgery [38,39] gluteal fat [40], and inguinal region [41,42]. In the current study fat was harvested from the inguinal area due to easy surgical access, the abundant collection and discrete scarring with no postoperative complications in any patients.

The use of PRP products especially PRGF technology has been extended to the treatment of OA in single and in serial repeated injections over time [5,10,31,32,43]. The autologous growth factors and proteins released from the fibrin scaffold may play an important role in the repair or regeneration of the damaged cartilage [5]. In the current study, a single injection of PRGF was opted for, in order to compare results without additional variables in the treatment regimen of the two groups.

In OA there is an overproduction of destructive substances and inflammation mediators that give rise to a balance in favor of articular cartilage catabolism [44]. The main problem with OA is pain, being the most common reason for consultation [10,44,45], but patients also present with stiffness, fatigue, walking limitations, discomfort, sleep disturbances, anxiety, and decreased quality of life [46]. Options for the treatment of OA vary considerably among authors [32] although biological therapies are a promising way of treating this disease, providing evident and lasting improvement that preserves tissue, improves the clinical signs and enhances articular function [47,48,49,50]. As mentioned, the objectives of treatment with regenerative therapies are the reduction of this parameter with consequent improvement of the functionality, using less aggressive therapies [51]. Both the application of aMSCs [22,52,53], and PRGF [32,54] demonstrate a decrease in the patient’s perception of pain. This effect is very important, as it has been shown that even after a joint replacement, 38% in hip and 53% in knee maintain some degree of pain one year after surgery [55]. Assessing the severity of lameness and pain due to OA is more challenging with canine patients than with human patients. The VAS scale has been used in previous studies [32,56], and are accepted methods for assessing pain. In this study, animals in both groups showed significant improvement in pain scores from the first month post treatment, perceived by both evaluators. So, with both therapies, one of the most important goals in the treatment of OA was achieved, the reduction of the pain experienced by patients in the affected area [10,57]. Furthermore, the beneficial effect achieved with both therapies was reflected in data provided by owners in the treatment satisfaction questionnaires, where both treatments achieved a high degree of satisfaction from the first month post-treatment and maintained until the end of the study.

OA is a degenerative disease that can affect all articular structures, causing joint degeneration with osteophyte formation, bone remodeling and alteration of periarticular tissues including the synovial fluid, joint capsule, subchondral bone, muscle, tendon and ligaments [58]. Among the methods of evaluation of this pathology, radiology is used as a simple technique for its availability and accessibility [59], although there is controversy as to its utility as a diagnostic method for the response to different treatments. It has been reported that OA in rabbits treated with mesenchymal cells derived from adipose tissue showed radiographic changes at 20 weeks post infiltration [60]. Contrary to a previous study [60], here, improvement in the signs of OA was not seen, however a progression of the signs was not seen either during the six month study, similar to those results observed in human [61] and veterinary studies where Silva *et al.* reported no significant differences in the radiographic exam between animals treated with PRP and control group after cranial cruciate ligament surgery [62]. The radiological analysis used in this study was inconclusive. One reason to explain these findings could be related to technical limitations for detecting and evaluating the progression of osteoarthritis in both groups, although radiographs were evaluated and scored in a multiple blind fashion. These results do not necessarily mean that changes did not exist at a cartilage structure level, but in order to demonstrate this, a magnetic resonance imaging (MRI) study should have been conducted to evaluate these structures in more detail or biopsies of the cartilage in affected joints should have been performed [63]. These complementary assessments were not performed due to the complications they implied for the owners.

The ROM in joints affected by OA decreases significantly [8,57], producing a decrease in functionality that has a detrimental effect on the ability to lead a normal life [1]. It has been observed that treatments with aMSCs [8,22] and PRGF [5,10,32] enhance the functionality of joints affected by OA which leads to an improvement in clinical signs, reducing lameness and providing notable recovery of previously limited walking and running activities [8,52]. In this study, enhanced joint mobility and functionality were observed in both of the treatment groups, although there is a significantly greater improvement in the aMSCs group at 6 months post treatment.

The effect PRGF has in the treatment of OA is due to the behavior of the platelet concentrate, acting as a scaffold which through the various growth factors promotes the stimulation of chondrogenesis, increases hyaluronic acid production, stabilizes angiogenesis and differentiation of the existing cells in the area treated [6,64]. Platelets are cells that contain many important bioactive proteins and growth factors (GFs) which are polypeptide substances, both soluble and diffusible, that regulate key processes in tissue repair, including cell proliferation, chemotaxis, migration, differentiation, and extracellular matrix synthesis [27,65,66]. The results of the current study coincide with those of numerous studies, both *in vitro* and *in vivo*, that have shown that PRP stimulates chondrocyte proliferation and matrix synthesis [67,68], and indicate that intra-articular injection of PRP prevents progression of osteoarthritis, improves articular function and reduces pain in the area [10,48,69]. However, with respect to the aMSCs, the effect is due to the contribution of culture expanded MSCs, which have the capacity for regeneration and differentiation into the various tissues involved, that is, new cells are provided that differentiate into cells at the injury site allowing for greater improvement in the area treated [70]. These types of cells express the potential to differentiate into multiple tissue lineages such as osteogenic and chondrogenic phenotypes [71,72] and have the ability to localize and participate in the repair of damaged joint structures, including cruciate ligaments, menisci, and cartilage lesions [21]. Additionally, aMSCs are known for their anti-inflammatory and immunomodulatory properties and for being a safe therapy without adverse effects [52,60]. In this study both groups increased ROM from baseline; although aMSCs had worse scores at the beginning of the study, dogs treated with aMSCs reported increased ROM at 6 months after treatment.

Thus based on the findings in this study, the therapeutic use of both PRGF and aMSCs are novel treatments that report goods results for overcoming the difficulties in regeneration of articular cartilage in OA while maintaining or improving the structure and joint function without the need for more aggressive techniques that can cause further damage to the organism. Mesenchymal cells are cell populations with molecular mechanisms capable of self-renewal and differentiation into various cell types in damaged tissue, repairing and supplying physiological functions in the affected joint, reducing pain, restoring joint function and delaying the onset of cartilage degradation [73,74,75]. In the case of PRGF, the proteins they contain have the ability to increase the proteoglycan and collagen synthesis by chondrocytes and regulate homeostasis and proper functioning of cartilage [75,76]. Notably, both PRGF and aMSCs improved the pathology at an articular level, with improvement to clinical signs and symptoms as well as to the structural damage caused by the disease [53,77]. Given that the animals in the current study showed significant improvement in all parameters studied but without obvious radiographic changes, it could be deduced that radiology may not be a sufficiently sensitive technique to assess the evolution of OA at the particular time points studied by the team of researchers in this parallel study [78].

In addition to OA, the beneficial effects of both treatments have also been described in Achilles tendon in a murine animal model and in facial nerve injury in a porcine experimental model, finding a synergistic effect on healing when PRP was used in combination with adult stem cells [79,80]. However, this synergistic effect was not demonstrated in a study on flexor tendons in sheep, where the group treated with MSCs showed an improvement in the composition and organization of the structural matrix, with increased expression of collagen I and cartilage oligomeric matrix protein, along with a decreased expression of collagen III, compared to the groups treated with PRP alone or in combination with MSC. The authors attributed the absence of the synergistic effect between the treatments to the possibility that the behavior and action exerted by each take completely different routes, or that protocols were not optimal for stimulation of this synergistic action [81].

Having demonstrated the therapeutic benefit of both treatments, one must take the advantages and disadvantages of each treatment’s clinical application into account. Although PRGF demonstrated a shorter duration of effect, it has the advantage of being substantially more affordable and far less invasive, and therefore simpler for ambulatory use. And it is important to highlight that PRGF treatment can be optimized with serial injections over time to obtain better results with respect to the duration of the effect [77].

Regarding the aMSCs, the main drawback is the need to perform minor surgery under general anesthesia for harvesting adipose tissue, with the advantage being the greater therapeutic benefit achieved. The possibility of optimizing this treatment is the creation of a bank of autologous cells, allowing for their availability without the necessity of further surgeries. This would bring the treatment into the outpatient clinical setting [76].

Minimally invasive methods for the treatment of OA in dogs are appealing to both veterinarians and pet owners, particularly when compared with surgical alternatives such as joint replacement.

The main limitations of this study include the lack of a placebo group, although recent studies have demonstrated that a single intra-articular injection of Saline Solution in OA dogs, has no effect in reducing pain or improving functionality [32]. It could be a very good option to have performed an MRI or a biopsy of the cartilage, and an arthrocentesis to collect synovial fluid at each visit. These complementary assessments were not performed due to the complications they implied for the owners. It is also true that the differences in the protocols makes it impossible to blind the treatment to the owner (dogs received a biopsy for the fat sample), but the evaluation of the dogs status and disease progression was performed by blinded physicians.

So, to fully demonstrate the potential of these two therapies in the treatment of OA, more double-blind randomized controlled trials should be performed as histological results could be very important for the future of these treatments. These studies should include the determination of biological markers of articular catabolism and anabolism in the serum and synovial fluid, and arthroscopic and histological evaluation of the cartilage.

## 3. Experimental Section

### 3.1. Animal Population and Randomization

The study was designed as a randomized, multicenter, blinded, parallel group, conducted in 3 veterinary hospitals. Dogs, male or female, with lameness of the legs were eligible for inclusion. A complete orthopedic and health history was collected as part of the screening and initial enrollment. Only dogs with hip OA were included in the study. A block randomization to either aMSCs or PRGF group was performed where an independent randomization coordinator, who was not responsible for determining the eligibility of the animals, included the animal and had no information about the dogs in the trial and no influence on the assignment sequence. All assignments were communicated by phone.

The following diagnostic criteria for patient selection were used: Animals suffering from degenerative joint disease in one or both hips, weighing more than 15 kg and being free of concurrent pathologies apart from OA. Prior to inclusion, a complete clinical evaluation (physical examination and vital signs were taken), complete hematology and serum biochemistry, endocrine and serology panel were performed. Orthopedic radiographs were reviewed to determine the degree of osteoarthritis in the affected area using the Bioarth^®^ scale (Bioiberica, S.A. Barcelona, Spain) for the hip joint, where the presence of OA was evaluated and evidenced by subchondral bone sclerosis, bone remodeling, osteophytes, or enthesophytes [82]. Once the animal completed the inclusion criteria, no treatments (non-steroidal anti-inflammatory drugs, analgesics, nutraceuticals, or adjunctive therapies) could be administered for two months. After this two month period, the animal was evaluated again as at baseline.

**Figure 5:**
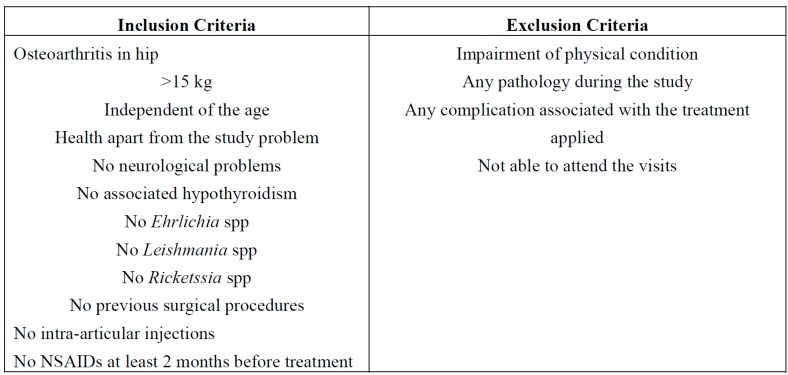
Inclusion and exclusion criteria.

Once the animal started the study, the exclusion criteria included any significant impairment of physical condition or functional status, whether as a result of the treatment, or for reasons beyond those previously mentioned (Figure 5).

All owners of participating dogs signed a written consent form where they were given relevant information about the study and agreed to the randomization procedure. There was one fixed price for all participants irrespective of the treatment given. The study was approved by the institutional animal care board at the Las Palmas de Gran Canaria School of Veterinary Science.

After this, dogs were assigned to one of the following groups:

○ aMSCs: single intra-articular injection of autologous aMSCs (DogStem^®^, Fat-Stem, Aalst, Belgium) (2 mL containing 30 million aMSCs) (*n* = 18).
○ PRGF: single intra-articular injection of autologous PRGF (PRGF-Endoret^®^, BTI Biotechnology Institute, Vitoria-Gasteiz, Araba, Spain) (2 mL) (*n* = 19).

As some animals were affected bilaterally, the total number of joints studied were 40 and 38 for the aMSCs and PRGF groups, respectively.

After the injection, the owners of the dogs were instructed to check for any sign of discomfort or pain, and were given instructions on the administration of cephalosporin (20 mg/kg/12 h, PO 7 days), meloxicam (0.1 mg/kg/24 h, PO, 3 days), and tramadol 82.0 mg/kg/12 h, PO, 24 h). Throughout the study the owners could use meloxicam as a rescue analgesic, to be recorded in writing for further assessment.

### 3.2. Treatments Applied

#### 3.2.1. aMSCs

The entire sample collection process was approved and certified by the dog owners with a signed informed consent. A biopsy of 20 g of subcutaneous fat tissue was collected from the inguinal region through a small surgical incision, and 120 mL of blood was isolated under aseptic conditions and processed with the DogStem^®^ kit (Fat-Stem, Aalst, Belgium). Immediately after sample collection, fat biopsy and blood were sent at 4 °C for cell isolation and amplification under current GMP conditions to the Fat-Stem Laboratory (Aalst, Belgium).

The adipose tissue was processed according to standardized Fat-Stem laboratory procedures and in accordance with the GMP laboratory regulations. The derived adipose tissue was digested enzymatically, washed and centrifuged several times to obtain a concentrate of cells. Subsequently, the mixture of cells was grown in a bioreactor environment with controlled temperature, oxygen and CO_2_ control. Parameters and conditions of growth were selected for the expansion of adult mesenchymal stem cells. Cultivation results showed that adjustments in cultivating conditions are necessary to optimize cell growth and to maintain the dog stem cells over several passages, e.g., autologous serum and/or additional growth factors. Different media settings have been tested on canine ASC during P1 to P4: Evaluation of the media was performed at 80% confluence of cells:

- Morphology: cells are plastic adherent, lack osmotic shrinkage, normal cell surface and fibroblastic shape.
- Ease of trypsinization: a single trypsinization.
- Color of the cultivation medium: Phenol red indicator is used to evaluate pH changes, which might affect growth of the stem cells.
- Proliferation time (doubling time): The population-doubling time (PDT) is defined as the time required for a colony area to expand twofold. Population doubling time calculates growth proliferation. PDT results are graphically visualized over passage time.
- Viability: Viability of the cells were quantified using trypan blue and cell counting methods were used.
- Cells were expanded and 30 million cells were used for clinical purpose.

Once the cells were amplified, the Fat-Stem Laboratory sent them in a 2 mL sterile solution containing 30 million aMSCs. In all cases, the laboratory certified cell quality. 

#### 3.2.2. PRGF

In the literature there are a many products named PRP, with different compositions and characteristics. In this study PRGF was used. It is the most advanced autologous Platelet-Rich Plasma system. It is based on the activation of the patient’s own platelets for the stimulation and acceleration of tissue healing and regeneration. It is 100% biocompatible, autologous, versatile and safe with control over its activation and use and with a simple, fast protocol: one 8 min of centrifugation and 20 min of preparation. Also, it does not contain leukocytes (avoiding proinflammatory activity), nor erythrocytes [25].

In all cases, blood was collected under aseptic conditions in 4.5 mL citrate tubes as necessary, and then centrifuged for 8 min at 460× *g*, then activated with 5% of its volume with 10% calcium chloride. The PRGF was applied immediately after its preparation and in none of the cases later than one hour.

### 3.3. Outcome Variables

All the patients were evaluated at baseline (T0) and 1 (T1), 3 (T3) and 6 (T6) months’ follow-up under sedation with dexmedetomidine (5 µg/kg), morphine (0.2 mg/kg) and midazolam (0.1 mg/kg). Each follow up assessed passive manual mobilization of the joint, degree of atrophy of muscles concerned with movement of the involved articulation, goniometric measurements of the range of movement, radiographs and analysis with the Bioarth^®^ scale (Bioiberica, S.A. Barcelona, Spain), a subjective patient pain assessment by the veterinarian and owner, questionnaire to owners on functional limitation of the animal, and satisfaction with the treatments applied.

#### 3.3.1. Pain Assessment

Global pain of the animals was assessed twice using a 0–100 mm VAS scale, where 0 mm signified “no pain”, and 100 mm marked “extreme pain” by both the owner and the investigator.

The patient pain assessment of disease status was performed by the owner answering the question: “Considering all the ways the arthritis affects your pet, please indicate the amount of pain that you think your pet is suffering by marking an (X) through the line”: 0–100 mm VAS scale: on the left “no pain”, and on the right “extreme pain”.


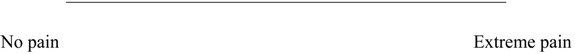

The investigator global assessment of the patient was performed with the question: “make a global assessment of the patient’s disease status by marking an (X) on the line below:”

0–100 mm VAS scale: left “no pain”, and right “extreme pain”.


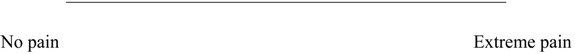


Once the postoperative treatment had finished, and if the owner perceived pain in their pet, meloxicam could be administered orally and then recorded in a diary for further investigation.

#### 3.3.2. Bioarth Assessment Scale [82]

This scale published for the assessment of osteoarthritis in hips was used for radiographic and functional assessment of each joint, in an independent manner [82]:

- Radiological assessment quantifies radiographic signs of osteoarthritis in canine elbows, hips and knees. Classifies the degrees of osteoarthritis into 4 categories 1: no signs of OA (0–2 points), 2: mild OA (3–8 points), 3: moderate OA (9–14 points), 4: severe OA (more than 14 points), based on a numerical assessment (0–3) of 7 anatomical points in the joint (limits of total score 0–21).
- Functional assessment evaluates the 3 basic functional parameters: functional limitation, joint mobility and muscle atrophy.
  ○ Functional limitation quantifies the weight-bearing or support of each extremity, changes in posture when standing still, signs of lameness when cold and when walking, endurance during walking and playing, endurance when going up stairs and strength limitations in small jumps (measurement scale: 0 to 23 points).
  ○ Joint mobility score is a summation of joint motion limitation, of the degree of flexion and extension of the joint studied (ROM: extension minus flexion) and pain when performing this assessment, with the goniometer (measurement scale: 0 to 7 points).
  ○ Muscle atrophy measured in centimeters, circumference of musculature at standard anatomical references. A measure ribbon was used taking three measurements and an average calculated. (Measurement scale: 0: no atrophy, 1: mild atrophy, 2: severe atrophy).

#### 3.3.3. Owner Satisfaction with the Treatment

When the animals started the study, owners were asked about the treatments received. Overall satisfaction on the treatment were recorded and then compared with the results obtained after 6 months of the infiltration with aMSCs or PRGF.

Outcomes in quality of life were also evaluated at baseline and at all the time points. These questions were scored on a five point Likert scale. For analysis, the responses were assigned numeric values 1 to 5, respectively.

○ very poor
○ poor
○ fair
○ good
○ excellent

#### 3.3.4. Safety Assessments

The nature, onset, duration, severity and outcomes of all side effects were assessed and documented at each visit. To evaluate the safety profile of the two treatments, all complications and adverse events were recorded with an accountability scale. 

#### 3.3.5. Requirement of Rescue Analgesia

Throughout the study, owners could use meloxicam (0.1 mg/kg) as rescue analgesia if they thought that their pets required it. The owner recorded the use of rescue medication daily.

### 3.4. Statistical Analysis

Data were analyzed with the SPSS 20.0 program for MAC (IBM, Madrid, Spain, 2012). Data were assessed for normality with the Shapiro-Wilk test, and non-parametric Kruskal-Wallis and Mann-Whitney *U* tests were used to compare quantitative variables at each follow-up time point between groups. A related-samples Wilcoxon Signed Rank Test was used to assess differences with baseline in each group. Crosstabs with contingency coefficient or Fisher’s exact test were used to evaluate the categorical variables as necessary. For VAS score, the percentage change before (baseline) *versus* after (1, 3, and 6 months) treatment was calculated for each group. A *p*-value < 0.05 was considered statistically significant.

## 4. Conclusions

The findings in the current study show that aMSCs and PRGF are safe and effective in the functional analysis at 1, 3 and 6 months; a significant improvement was provided, reducing dogs’ pain, and improving physical function with respect to basal levels in patients with hip OA. Compared to PRGF, aMSCs showed better results at 6 months, though it is a more aggressive treatment when considering its procurement. Both treatments should be considered in the treatment of dogs with hip OA.
